# Inferring haplotypes and parental genotypes in larger full sib-ships and other pedigrees with missing or erroneous genotype data

**DOI:** 10.1186/1471-2156-13-85

**Published:** 2012-10-10

**Authors:** Carl Nettelblad

**Affiliations:** 1 Division of Scientific Computing, Department of Information Technology, Uppsala University, Box 337, SE-75105, Uppsala, Sweden

**Keywords:** Haplotyping, Phasing, Genotype inference, Nuclear family data, Hidden Markov models

## Abstract

**Background:**

In many contexts, pedigrees for individuals are known even though not all individuals have been fully genotyped. In one extreme case, the genotypes for a set of full siblings are known, with no knowledge of parental genotypes. We propose a method for inferring phased haplotypes and genotypes for all individuals, even those with missing data, in such pedigrees, allowing a multitude of classic and recent methods for linkage and genome analysis to be used more efficiently.

**Results:**

By artificially removing the founder generation genotype data from a well-studied simulated dataset, the quality of reconstructed genotypes in that generation can be verified. For the full structure of repeated matings with 15 offspring per mating, 10 dams per sire, 99.89*%* of all founder markers were phased correctly, given only the unphased genotypes for offspring. The accuracy was reduced only slightly, to 99.51*%*, when introducing a 2% error rate in offspring genotypes. When reduced to only 5 full-sib offspring in a single sire-dam mating, the corresponding percentage is 92.62*%*, which compares favorably with 89.28*%* from the leading Merlin package. Furthermore, Merlin is unable to handle more than approximately 10 sibs, as the number of states tracked rises exponentially with family size, while our approach has no such limit and handles 150 half-sibs with ease in our experiments.

**Conclusions:**

Our method is able to reconstruct genotypes for parents when genotype data is only available for offspring individuals, as well as haplotypes for all individuals. Compared to the Merlin package, we can handle larger pedigrees and produce superior results, mainly due to the fact that Merlin uses the Viterbi algorithm on the state space to infer the genotype sequence. Tracking of haplotype and allele origin can be used in any application where the marker set does not directly influence genotype variation influencing traits. Inference of genotypes can also reduce the effects of genotyping errors and missing data. The cnF2freq codebase implementing our approach is available under a BSD-style license.

## Background

Inference of haplotypes, or phasing, from genotype and pedigree data can be useful in several ways. For traditional linkage analysis, including QTL mapping, knowledge of haplotypes can help in producing a more correct analysis of linkage accurately tracing individual recombination events, and thus higher statistical power. Phase data can also be used directly or indirectly for genome-wide association studies (GWAS), for example by using knowledge of phase for a sparse marker map within a local population as the basis for dense genotype imputation based on reference populations [1].

In this article, we focus on reconstruction of haplotypes where pedigrees are known and assumed to be reliable. In such populations, full genotypes can be missing in ancestral generations, for some or all individuals. This situation is especially frequent for species with long generation times, where older historical records have to be used. In wild mammal populations, the paternity of a litter might also be unknown or unsure. Inference of haplotypes in sib-ships with some or all parental genotype information missing has therefore been given more attention recently [2,3].

Our approach to resolving haplotypes and missing genotypes is based on repeated local analysis of focus individuals and their immediate ancestors, representing further relations by introducing a parametrization (called skewness) of phase in each marker. This can be contrasted against earlier models trying to create a global representation of the full pedigree [4]. We have earlier described and demonstrated a less refined version of this approach with simulated datasets [5,6], with an underlying assumption that genotypes are static. We now extend the model by treating the genotypes themselves, previously defined as fixed and definite, as model parameters (sureness values) that can be optimized (fitted) based on the data. Using the model and a specifically adapted optimization algorithm, we demonstrate reconstruction of completely missing parental genotypes from offspring genotype data with high accuracy.

Our model is suitable for large families, a large number of generations and a large number of markers, making it a versatile tool in all contexts where the pedigrees are known and haplotypes or inference of missing genotypes would be useful. Our methods are also applicable to any pattern of missing ancestral genotype data, e.g. only one parent genotyped, only a subset of microsatellites genotyped or completely missing genotype data for both parents, allowing higher accuracy and new types of analysis to be performed on a wide set of experimental datasets.

## Methods

Our model is a specialized Hidden Markov Model (HMM). In general, an HMM is defined by its states and transitions between states [7]. The use of HMMs for tracing haplotypes has a long history. In fact, certain aspects of the approach presented here are shared with the original presentation of MAPMAKER [8] (the Lander-Green algorithm). Efficient implementations of related models have also previously been made with the explicit goal of extracting haplotypes from genotype data [4,9]. However, many descriptions either describe only the model without attention to important algorithmic aspects in their implementation, or do not clearly separate computational details from the mathematical representation of the statistical models.

As much work in the field has been incremental, we therefore here present our model in full, followed by a description of relevant aspects of the optimization algorithms used to fit the model in practice, i.e. adapting the skewness and sureness parameters based on the observed data. In addition, since the results of our experiments are compared against results from Merlin [4], we specifically mention similarities and differences in the modelling compared to that tool. The approach described is similar to our earlier work [5,6], but the description of the model and algorithm is more thorough. The handling of genotype uncertainty represented as sureness parameters is completely novel, while other aspects have also been improved.

### The building blocks of an HMM

An HMM consists of a set of *states*, allowed *transitions* between states and *emitted symbols*[7]. The hidden aspect of the model is that the state sequence is generally assumed to be unknown (and hence also the transitions), while the emitted symbols are possible to observe. One out of many popular bioinformatic applications for HMMs is motif-finding, where the state is an idealized (hidden) description of the protein structure. Transitions would describe the possible orderings of such structures, while nucleotides or amino acids would form the emission alphabet.

We will present an HMM for haplotype determination in pedigrees. The model is versatile, based on the observation that when phase information is available, the phasing of a single non-phased individual can be performed using only a local pedigree with the closest ancestors. We therefore parametrize uncertainty in phase as well as specific alleles by the introduction of parameters that we choose to call *skewness* (*γ*_*im*_) and *sureness* (${â}_{\mathrm{im}}$).

Many HMMs tend to be time-independent. This means that the emission and transition probabilities are defined solely by the state of the model, no matter at what position in the sequence that state is found. However, time-dependent HMMs, where the emission and transition probabilities are dependent on the state as well as the position (marker number), can also be powerful, especially when the emitted symbol structure is complex. The model we are using is of this nature. The emitted symbols consist of marker values, or specifically, pairs of binary (for biallelic markers) or other discrete values (for e.g. micro-satellites), which are naturally different for different markers. The Markov property is still maintained, since the probabilities are independent of the states in other markers.

### Choice of state space

The states that result in emissions in our model consist of *haplotype inheritance pathways*, indicating which of the two haplotypes in a diploid individual that was transmitted, at a specific position, to a specific individual offspring. A state realization for two parents and an offspring (a trio) can for example be “chromatid 1 from father, chromatid 2 from mother”.

**Figure 1:**
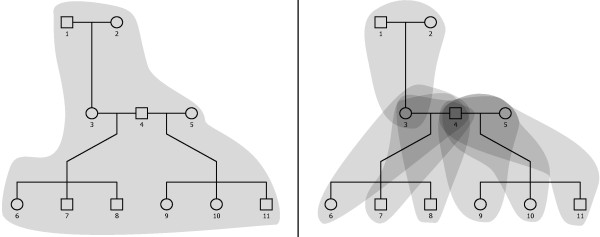
**State space decomposition.** A pedigree for a small set of half-sibs, including parents and one set of grandparents known. In Merlin (left) [4], all meioses, i.e. all allele transmission, are covered by the state representation in a single stochastic process. In our model (right), on the other hand, the pedigree is decomposed into a series of focus pedigrees, with one individual and its immediate ancestors *k* generations back. Here, *k*=1. Some individuals in this pedigree then appear in up to 6 unique focus pedigrees. Parameter values representing phase and unknown genotypes are shared among all focus pedigrees. Thus, global information on allele transmission is inserted into the individual focus pedigrees, while avoiding the exponential explosion in the size of the state space resulting from explicitly keeping track of all meioses at once, as is done in e.g. Merlin.

The state representation forms a crucial difference between our work and that represented by e.g. Merlin [4]. This difference is illustrated in Figure 1 (original pedigrees drawn using the R package kinship2[10]). Merlin represents a full clique of connected individuals (i.e. a possibly multi-generational family) as *one* stochastic process, while our model decomposes the clique into several separate *focus pedigrees* of limited size and hence multiple stochastic processes. The state in Merlin consists of the global haplotype inheritance pathway tree, going back from every descendant to the original founders. In a text description, a state within the pedigree shown in Figure 1 could read “chromatid 1 from founder **1** to offspring **3**, chromatid 1 from founder **2** to offspring **3**, chromatid 2 from offspring **3** to offspring **6**, chromatid 1 from offspring **3** to offspring **7**..” . As each meiosis includes a binary choice, the cardinality of the total theoretical state space becomes approximately 2^*N*^, where *N* is the cardinality of the set of related individuals. Many of these states are unlikely as they would be inconsistent with the observed marker values. This sparseness property forms the basis of the sparse gene flow trees used in the implementation of Merlin. Since the state models the full tree of meiosis events, haplotypes can be determined in one single run or iteration over the data, using e.g. the forward-backward algorithm, without any need for iterative refinement.

Our model, on the other hand, is inherently local, but using an iterative approach to still share global information from all parts of the pedigree. A complex pedigree is viewed as multiple instantiations of the smaller problem of a single offspring individual and its ancestors *k* generations back. For practical purposes *k* is restricted to 1 or 2. Already *k*=3 would lead to 16,384 states, as the number of states grows exponentially on the number of ancestors, which itself grows exponentially on the number of generations.

It is important to observe that in the case of informative markers phasing can be solved for small local pedigrees straightforwardly applying the Mendelian laws. Thus, in informative markers, our model is able to resolve the haplotypes, with no additional input. If both parents are heterozygous in a marker, and thus that marker in itself is not informative, some states will still be more likely than others, as only some would correspond to emitted symbols matching the observed data. If parental genotype phase and linkage to neighboring markers are known, only a few states will have a non-negligible probability. Those states will indicate the correct haplotype set in the offspring.

When all focus pedigrees are analyzed in the first iteration, using HMM algorithms, the state sequence will only be uniquely defined in those regions where markers are fully informative. We then iteratively refine parameters to infer a consistent haplotype resolution in all markers step by step, not only the most informative cases.

The description so far leaves no specific parameter to refine in order to determine and represent haplotypes. For this purpose, we introduce the two additional parameters per marker per individual, *skewness* and *sureness*. The purpose of introducing these is to carry relevant information between focus pedigrees, as well as being useful indicators of the true genome structure in their own right.

### Determining emission probabilities from states

**Figure 2:**
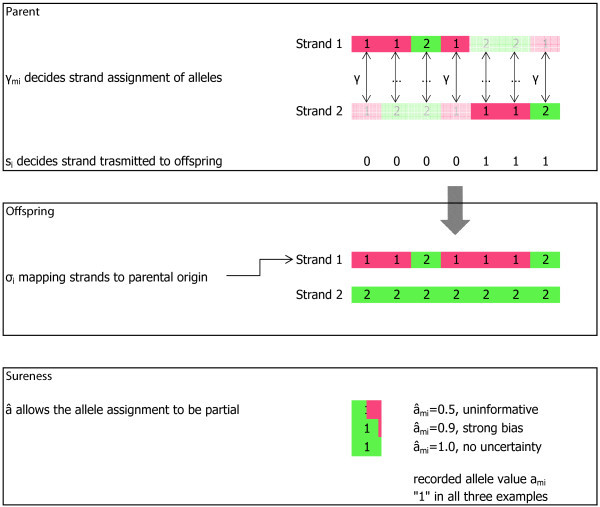
**Illustration of model.** Panels, from top to bottom: 1) In a focus pedigree, the skewness parameters *γ*_*mi*_ per marker per individual determine chromatid assignment of the recorded alleles. A state bit *s*_*i*_ per individual in the pedigree (except for the focus individual) determines which chromatid was transmitted to the offspring. Here, we see a recombination after the fourth marker in an imagined state sequence. The alleles listed in the figure should be interpreted as the actual emitted symbols. 2) In every non-founder in the focus pedigree, the shuffling flag *σ*_*i*_ maps the two chromatids to parental origin. Here, chromatid 1 is defined as originating from the parent shown in the panel above. 3) By introducing sureness values â, any recorded allele value can be treated as not fully definite. In an iterative refinement, the genotypes can be inferred, going from low information to an almost definite assignment. This is useful both for handling genotyping errors and for inferring missing genotypes.

We will now present in detail how to compute the emission probabilities, first without our added parametrization, and then including skewness (*γ*_*im*_) as well as sureness (${â}_{\mathrm{im}}$) parameters. An illustration of critical aspects of the model structure, and the effect of our added parameters, is found in Figure 2.

Without the parametrization, the emission probabilities for all permissible symbols for a state are identical, while the rest are zero. A permissible symbol would in this case consist of a configuration of ordered marker pairs that is internally consistent with the inheritance pathway defined by the state. If the state includes chromatid 1 in *A* being transmitted to chromatid 1 in *B*, which in turn is transmitted to chromatid 1 in *C*, then the emitted pairs for *A*, *B*, and *C* need to include the same allele in a correct chromatid position.

The state *s*∈*S* can thus be represented as a binary string of *N*−1 digits, each stating which of the two chromatids that was transmitted from an ancestor to its offspring in the current focus pedigree. The focus individual itself has no offspring in the pedigree, and thus no specific state bit. In addition, we introduce a chromosome-wide *shuffling flagσ* determining the parental identity, mapping the two distinct chromatids in an individual to its actual parents. This flag includes one binary value per non-founder individual within the focus pedigree. Depending on the realization of the model, *σ* can be kept fixed (e.g. all zeros), or the full model can be evaluated over the chromosomes for all possible assignments of *σ*. Unless phase is specified explicitly in some way, the emission probabilities will be symmetrical for different choices of *σ* (modifying the state accordingly), as the chromatid assignment is then arbitrary.

The emitted symbols can be seen as a string with two (ordered) marker symbols per individual (2*N* in total). Alternatively, they can be represented as *N* binary digits, if we assume that the observed marker values are correct, and we only need to specify the chromatid assignment for the two observed values per individual (e.g. *xy* or *yx*). The latter description is more amenable for analysis, so we will describe emitted symbols *e*∈*E* as such strings of *N* binary digits. Note that for any homozygous marker, the two orderings are outwardly identical.

In summary, the actual allele being transmitted into a specific offspring individual is thus dependent on up to three things, each being a binary flag; the state *s*, the emitted symbol *e*, and the shuffling flag *σ*. We define an operator *Q* as applying an exclusive or operation on these three, or equivalently, $\left(s_{i}^{\prime }=Q_{i}(s_{i},e_{i},\sigma_{i})=\left(s_{i}+e_{i}+\sigma_{i}\right)\;\mathrm{mod}\;2\right)$ (substituting 0 for those individuals *i* where the relevant terms are not defined). The list of alleles transmitted $\left(s_{i}^{\prime }\right)$ can be concatenated into a string *s*^*′*^.

If we call the set of permissible allele transmissions *S*^*′*^, then the non-normalized emission probability function without parametrization for phase for marker *m* in the focus pedigree *P*, *E*(*e*,*s*,*σ*,*m*,*P*), can be expressed as:

(1) $$E(e,s,\sigma ,m,P)=\left\{\begin{array}{cc} 1\; & s^{\prime }\in S^{\prime }\\ 0\; & s^{\prime }\notin S^{\prime } \end{array}\right)$$

This definition of *E* would hold for Merlin and our model alike, with the noted differences in the size of the pedigree included in the state space representation. Based on this definition of *E*, we can extend it to include our added parameters, skewness and sureness.

### Skewness

We have previously proposed [6] that the phase can be successfully parametrized as a scalar variable per marker per individual, which we call *skewness*. This parameter is shared between different focus pedigrees where the same individual appears. Thus, in an iterative updating scheme, information from different focus pedigrees will be fused to compute the update, and then distributed to all analyses in the next iteration. We use *γ*_*im*_ to denote the skewness for marker *m* in individual *i*.

The skewness determines the relative probability for ordering the unordered pair *xy* as *xy* (skewness 0) or *yx* (skewness 1). That is, the skewness states whether the true, biological, ordering of the marker values matches the order in which they were more or less arbitrarily recorded in the genotype data, or if it is the opposite ordering. If the ordering is non-determinable, the skewness should be 0.5. Conflicting data, where different offspring individuals make different haplotype resolutions likely in their common ancestors, could result in some other fraction.

The emission function when taking the set of skewness values *Γ* into account can be expressed as:

(2) $$E_{\gamma }(e,s,\sigma ,m,P,\Gamma )=E(e,s,\sigma ,m,P)\overset{N}{\underset{i=1}{\prod }}\left(1-|e_{i}-\gamma_{\mathrm{im}}|\right)$$

Taking skewness into account thus can be described as an independent filter on the non-phased emission probabilities.

### Sureness

While skewness can separate phase when the alleles are known, optimizing the skewness assignment will not help if *both* alleles in some marker in an individual are unknown. To handle the assignment of unknown alleles in a similar way, by gradual refinement, eventually converging to the correct assignment, we introduce the *sureness* parameter.

The description so far has treated the actual observed marker values as completely fixed. This is not fully appropriate for several reasons. Genotype information can be incorrect or inconclusive. To cope with genotyping errors, a small non-zero emission probability is allowed in some models for configurations that would not be permissible (outside of *S*^*′*^). This is not the case in Merlin, as a small probability for any state would break the sparseness assumption which is crucial to making Merlin efficient. However, to handle systematically incomplete information or error-prone genotype data, a more thorough representation of this lack of information is needed.

We call the total set of possible alleles for each marker *A*_*m*_, and the two alleles in each individual *a*_*im*_(0,1). The string *s*^*′*^ representing the allele configuration used in eq. 1 can thus be rewritten to be independent of the specific alleles observed by constructing *a*^*′*^ as the concatenation of *a*_*im*_(*s*^*′*^*i*), analogous to constructing *s*^*′*^ by concatenating *s*_*i*_. Call the set of permissible allele configurations in this form *A*^*′*^ and redefine *E* as *E*(*e*,*s*,*σ*,*m*,*P*,*A*):

(3) $$E(e,s,\sigma ,m,P,A)=\left\{\begin{array}{cc} 1\; & a^{\prime }\in A^{\prime }\\ 0\; & a^{\prime }\notin A^{\prime } \end{array}\right)$$

The indicator function for set membership of *a*^*′*^ in *A*^*′*^ can be rewritten as a function ${Ā}^{\prime }$ of (*e*,*s*,*σ*,*m*,*A*):

(4) $$E(e,s,\sigma ,m,P,A)={Ā}^{\prime }(e,s,\sigma ,m,A)$$

Here, *a*_*im*_(0,1) is defined as a scalar, which can alternatively be represented as an indicator vector within the set of possible alleles, with a single 1 value and the rest being 0. We add the sureness parameter allowing the singular 1 to take another value, representing some level of uncertainty of the genotype (see Figure 2). Just like we have *a*_*im*_(0,1), we then also have ${â}_{\mathrm{im}}(0,1)$, which represents the sureness of the allele specified by *a*_*im*_ being correct, and $1-{â}_{\mathrm{im}}(0,1)$ for any other allele. Rather than there being a single canonical $\left(a_{i}^{\prime }\right)$ for a specific configuration (*s*_*i*_,*e*_*i*_,*σ*_*i*_), there are instead different probabilities for different alleles in $\left({â}_{i}^{\prime }\right)$. Likewise, ${â}^{\prime }$ also becomes a multi-dimensional probability space for all possible allele configurations, although with most of the probability mass centered on *A*^*′*^ (assuming all surenesses being close to 1).

The corresponding entity to the simple indicator function for set membership in *A*^*′*^, ${Ā}^{\prime }$, then becomes:

(5) $${\hat{Ā}}^{\prime }(e,s,\sigma ,m,A,Â)=\underset{\alpha \in A}{\sum }{â}^{\prime }(\alpha )$$

This results in another new expression related to *E*:

(6) $$Ê(e,s,\sigma ,m,P,A,Â)={\hat{Ā}}^{\prime }(e,s,\sigma ,m,A,Â)$$

Finally, if we use the sureness-aware Ê, we can also construct the skewness-sureness-aware ${Ê}_{\gamma }$, using the same filtering definition as in eq. 2:

(7) $$\begin{array}{l} {Ê}_{\gamma }(e,s,\sigma ,m,P,\Gamma ,A,Â)=Ê(e,s,\sigma ,m,P,A,Â)\\ \;\times \overset{N}{\underset{i=1}{\prod }}\left(1-|e_{i}-\gamma_{\mathrm{im}}|\right)\; \end{array}$$

It is natural to fix â values to at least 0.5 (otherwise, another allele than the one indicated by *a* would be the most likely one). By the introduction of sureness, even completely untyped individuals can be entered into the analysis. Iteratively, their genotypes can be inferred and the sureness values can converge.

In our previous work, only skewness was included, limiting the flexibility in handling incorrect or missing genotypes. A parameter configuration including skewness, but not sureness, on the other hand, would not lend itself to simple optimization. Our treatment of sureness requires the two alleles in a marker to be distinguishable, and it is only the introduction of skewness that achieves this goal in the model structure.

### Transition probabilities

Between different markers, state transitions can take place. As a Markov process does not have any memory, the most obvious model of recombination will match Haldane’s mapping function [11], with no recombination interference. The recombination fraction in a single meiosis between markers *m* and *m* + 1 can be represented as *r*_*m*_, supposedly in the range [0,0.5), as normal linkage would not be upheld otherwise.

The total transition probability between states *s*_0_ and *s*_1_ (written as a sequence of binary digits) can thus be computed as:

(8) $$T(m,s_{0},s_{1})=\overset{N-1}{\underset{i=1}{\prod }}|1-|s_{0i}-s_{1i}|-r_{m}$$

The upper limit of the product excludes the last individual, the focus individual itself, as we are not tracking any meioses, and hence no state, from that individual. Although we have here assumed *r*_*m*_ to be identical for all individuals in the population, it is fully possible to generalize this in order to account for sex-specific or other patterns in recombination probabilities, as done in other work using HMMs for genotype or haplotype analysis [12]. The values *r*_*m*_ also do not need to be specified *a priori*, but can be part of the parameter set being iteratively optimized. However, for the Markov model to hold, the marker *order* should be pre-defined and identical for all individuals. Sex-specific recombination probabilities as well as iterative updates of them are included in our actual implementations of the model.

### Summary of model

In our model, a set of different focus pedigrees, each centering on a single *focus individual* and its ancestors *k* (*k*=1,2) generations back are analyzed separately. By including a set of optimizable parameters, including *skewness*, *sureness*, and possibly mapping distances between markers, information can be shared between all focus pedigrees in an iterative manner. Haplotype resolutions that are likely or unlikely due to the Mendelian inheritance of alleles and genetic linkage in one focus pedigree can influence those parameter values. Thus, all available information can be taken into account, while not tightly coupling the individuals into a single Markov process (as is done in e.g. Merlin). Doing the latter can result in an exponential explosion of the number of states, severely reducing the practical applicability of the model.

As our parametrization is based on scalar probabilities, rather than binary values, deterministic optimization methods can be used. This can be contrasted against models where only non-deterministic Markov-Chain Monte Carlo algorithms are used to fit the models in practice, e.g. [13,14].

### Method of model optimization

Actually finding optimal parameters for an HMM is not a trivial task. In fact, it is generally established that for all but the most simple cases, one can not expect to find a global optimum, nor is it necessarily tractable to quantify the quality of a local one. Our optimization method is based on the generally established approach of the Baum-Welch algorithm [15], itself a specific version of expectation-maximization [16] for HMM parameter optimization. The objective function that the algorithm attempts to maximize is the likelihood of the observed data, given the model. This can be understood by the fact that HMMs are *generative* by their nature. Although they are frequently used to analyze observational data, what they prescribe is the (hidden) stochastic process generating such observations. Adapting the parameters to maximize the likelihood means that the model of the generating process is adapted to match the observations.

The practical application of the Baum-Welch algorithm involves counting the frequency of different events, such as emitted symbols (conditioned on state) and transitions (between states). The expressions for the frequencies are parametrized by probabilities, and it can be shown that the likelihood is consistently improved if the new value for a probability parameter is chosen based on the posterior value for that probability based on a previous analysis. Again, note that this consistent improvement does not mean that the method in general will converge to a *global* optimum in likelihood.

In our application, we have multiple observations, as each focus pedigree consists of one separate realization of the process. The total likelihoods for multiple independent Markov chains are however equivalent to the product of the likelihoods for the independent chains, which in turn would be equivalent to a uniform state transition probability distribution between the last position in one chain and the first position in the next one. Thus, since it is proven that the Baum-Welch algorithm optimizes the parameters for a single observation chain, it also by definition optimizes the parameters for multiple chains in the same model. That means that parameter updates from multiple observations can simply be combined linearly, with consistent improvements in the compound likelihood for each iteration.

However, when *k* (the number of generations tracked) exceeds 1, updates for the non-immediate ancestor generations need to be weighted. If we consider the sample pedigree in Figure 1 (although that figure illustrates *k*=1), there are two meiosis events resulting in individual 3. Individual 3 in turn also has multiple offspring, but they are all observations showing different aspects of the same stochastic process representing the meioses generating individual 3. The updates to individuals 1 and 2 from any descendant pedigree should be equivalent. That means that if there would be siblings to individual 3, no matter what number of offspring they have, the total update from a child and its children to the grandparents should be weighted equally. As individual 3 has three offspring, the updates to individuals 1 and 2 from 3 and each of its children should therefore be divided by four, in order to be weighted equivalently to a child with no offspring on its own.

Although generally simple and based on standard algorithms, our implementation has certain properties that merit specific mention, as we have found them critical to efficient convergence not only to *some local optimum*, but to one of acceptable quality consistent with biological expectations. The general motivation behind many of these concerns and their solutions is the fact that compared to many HMM applications, our models could be perceived as over-parametrized, as we are representing a location-dependent single realization of a stochastic process, resulting in specific parameters per marker and per individual.

Specifically, we will discuss the issues of:

* Initialization values - as the approach can converge to a local maximum, the choice of initialization values for parameters can greatly influence convergence, or introduce bias.

* Skewness updates - dampening is needed to fully allow global information to propagate to all focus pedigrees and across markers. Care needs to be taken when weighting updates from multiple generations.

* Inversions - the updates in the Baum-Welch algorithm work on a single-parameter basis, but our parameter representation could induce errors that are global in nature. This issue can be resolved by testing whether likelihood is improved by inverting all values downstream of some location.

* Sureness updates - sureness values need to be capped at 0.5, again reaffirming the need for a good choice of initialization values, as well as dampening during the update iterations.

* Recombination fraction updates - if the generated mapping distances are to be used more generally, the recombination fraction in marker stretches where the specific location cannot be determined should be averaged out, rather than allowing the full recombination mass to converge on a single marker gap.

#### Initialization values

One important aspect in initializing the HMM, and expectation-maximization optimizations in general, is to avoid total symmetry. For example, it might seem reasonable to initialize all skewness values to 0.5. However, bar numerical instability, with no absolute reference, the skewness values would then never converge. A random initialization for parameters will (almost certainly) avoid such a stalemate, but the bias induced can be hard to assess. We have therefore tried as consistently as possible to maintain biologically and mathematically justifiable starting values, reflecting the (lack of) prior information.

The case where this needs to be explored in more detail is that of the skewness values. We choose to initialize the first heterozygotic marker on each chromosome in each individual to 0.

When handling genotype uncertainty in the offspring genotypes, the initial heuristic inference of allele and sureness values needs to reflect this fact. The heuristics used are based on Mendelian rules, and given a non-zero error rate and enough individuals, all markers will be resolved as heterozygous. Therefore, the sureness initialization needs to represent the count of individuals supporting the inferred allele. We suggest summing the logit values for the genotype error rate based on the sureness values of all offspring supporting a specific interpretation, and then dividing by a fourth of the total number of offspring, which is the count expected given Hardy-Weinberg equilibrium. In other aspects, the heuristics are similar to the ones used by e.g. GridQTL [17] when computing line origin probabilities.

#### Skewness updates

There is a definite risk that the updates suggested by the Baum-Welch algorithm will vary drastically, or even start oscillating, given the strong dependencies between separate focus pedigrees. For this reason, we have modified the Baum-Welch updates of skewness in order to make the method converge appropriately in practice, i.e. given the effects of floating-point inaccuracy. In addition, compared to ordinary HMMs, the binary nature of the phase “events” means that either *γ*_*mi*_ or 1−*γ* will by necessity be included for some skewness *γ*_*mi*_ in all focus pedigrees where the corresponding marker and individual appears. Even a comparatively small absolute change in *γ* can then exert a considerable influence on the likelihood.

For numerical reasons our implementation records the logarithm of the frequency ratio between the two phasing realizations from each focus pedigree. These are summed and normalized. Due to the property where the likelihood will always include either *γ* or 1−*γ*, a logit transform is applied. In the logit space, the updated skewness is dampened by a factor *d*_*γ*_, chosen to be 0.1. Especially when sureness is optimized at the same time, the dampening ensures that information is propagated before skewness values become too fixed.

It should be noted that even in double-precision floating point arithmetics, the “opposite” interpretation of say 15 consecutive markers when *γ*=0.1 will vanish and become equal to the machine epsilon (≈10^−15^). Without dampening, longer range phase dependencies might not start influencing the skewness updates before they are hidden by numerical inaccuracy, especially when sureness updates are performed simultaneously.

In addition, a capping is also included for the maximum relative change in the ratios $\left\{\frac{\gamma }{1-\gamma },\frac{1-\gamma }{\gamma }\right\}$. The capping constant controlling the maximum change, *ρ*_*γ*_, has been fixed at 3 when sureness updates are not included, but decreased to 1.6 when optimizing sureness as well. The reasoning behind introducing such a constant is similar to the one for *d*_*γ*_. Having both constraints, rather than just one of them, allows both to be chosen less conservatively, ensuring faster convergence. Of the two, *ρ*_*γ*_ is the more problematic, as the overall direction of the update vector is changed by capping the most drastic changes. The effects from *d*_*γ*_ are a more classical dampening. Despite this, introducing *ρ*_*γ*_ is important in cases where the first few iterations exclusively suggest one single interpretation, before sureness updates and other skewness values have begun to settle, and so modifying the picture.

#### Inversions

Updates of the parameters, skewness as well as sureness, are done on a per-marker basis. The posterior probabilities are computed, given all existing parameter values. If there is limited information in some marker region, the skewness values can be “flipped”. That is, a local region converges to values opposite of what they should be, in relation to the reference marker. This issue can appear more easily in founders, as there is no inherent haplotype reference based on parental genotypes for those individuals. However, not all errors in a model fit can be properly resolved by a per-marker update.

During the iterative Baum-Welch update process, a single marker on the border of an inverted region “sees” influence from the flipped region as well as the correct reference, but the two flanks will pull the *γ* update in different directions. There is then no way to improve the likelihood by modifying the skewness of a single marker locally.

If two parents are both missing genotypes, such an error in skewness in the offspring can also result in inferred genotypes (alleles *a* as well as sureness values â) possibly being flipped between the parents, as well.

Although this condition will not be handled by the conventional Baum-Welch updates, thanks to the structure with the state *s*, the shuffling flag *σ* and emitted symbol *e* all being binary flags, it is not computationally demanding to compute the resulting likelihood from an arbitrary inversion of skewness values as well as switching genotypes at any marker and downstream thereof in a focus pedigree.

For full sib families, or when only considering flips involving a single individual (i.e. not moving genotypes between parents) it is also relatively simple to combine the likelihood contributions from different pedigrees. In this way, we can take into account a selected set of updates that are not confined to a single marker, effectively eliminating the problem of inversion bubbles without influencing the algorithmic complexity. Thus, this type of update can scale well even as the marker count is increased (itself making local inversions more likely).

Our implementation compiles a list of all inversion configurations at all markers that would render a total increase in likelihood. From this list, a set of flips incurring the greatest relative likelihood increases, while not overlapping each other, is chosen. No individual can be included in multiple flips in the same iteration. If this condition would not be included, in many cases several flips would be chosen in neighboring markers, as they all, individually, would improve likelihood. The end result would be that they would neutralize each other, or create patterns of oscillatory behavior between iterations.

The general problem of finding a non-overlapping and consistent set of flips involving genotypes in pedigrees beyond full-sib families translates into a more complex graph matching problem, similar to 3-SAT. This is an avenue for future work. The implementation of inverting skewness for single individuals has previously been tested in complex multi-generational pedigrees [5,6].

#### Sureness updates

The updates on sureness and accompanying alleles (â, *a*) are a rather straightforward application of the Baum-Welch algorithm, but special care needs to be taken to allow sureness and skewness to work in tandem, separating heterozygotes from homozygotes. In order for skewness updates to work, the two alleles in a marker need to be distinguishable by differences in *a*_*im*_ or ${â}_{\mathrm{im}}$. A completely standard sureness update algorithm would distribute the probability of the two alleles equally on the two positions in a marker. If there is some slight over-representation of one allele in the data, the result would be that many heterozygotic markers would be inferred as homozygotes with rather low sureness values for both chromatids.

Our current solution is two-pronged, involving the initialization as well as the update process. First, missing genotypes are inferred heuristically based on alleles appearing in offspring. This pre-inference step gives an initial substrate for the true genotypes, e.g. both parents carrying a 1 allele, fixing that allele in one of the two chromatids. When this initial separation between the two alleles has been introduced, the skewness and sureness updates in tandem will find which parent might be carrying a 2 allele etc. A dampening is also used for sureness updates to avoid oscillatory behavior between the updates of the two closely related sureness values in each marker in each individual.

The current implementation only handles sureness updates in first-level parents found in the focus pedigrees, although the concept is not inherently limited to that case in any way.

#### Recombination fraction updates

Due to numerical inaccuracy, as well as the shifting interpretations due to other parameter updates, a stretch of markers *m*_1_…*m*_2_ where there are no (or very few) recombination events in the dataset can be represented in such a way that most of the recombination fraction for the whole region is “absorbed” in a single $r_{m^{\prime }}$, where *m*^*′*^ can be any marker in that range. The other *r* parameters then converge to 0.

While compatible with a single set of observations, the presence of such a region does not result in a robust map that would properly account for the possible interpretations, especially if the purpose is to have a general map applicable to other samples from the same population. If there are indeed a few recombination events present that are only recognized when the phases converge, those are also more easily detected if the recombination fractions have not already approached an extreme configuration.

For this reason, we have implemented an explicit test verifying whether the total likelihood is affected by distributing the recombination fraction evenly between every set of pairs of neighboring markers. In cases where there is no information either way, that test will succeed. When that happens, the existing *r*_*m*_ values are shared between the two markers, irrespective of their previous values. Just like the inversion testing described previously, this check can be performed using the existing data structures from the forward-backward algorithm and Baum-Welch training, thus incurring no change in algorithm complexity and only a very slight penalty to runtime.

### Parallel execution

Compared to a model where the meioses for all related individuals are tracked in a single state space, our model approach and optimization algorithm are far simpler to run in parallel on modern computing architectures. The analysis of each focus pedigree can be performed independently in a separate thread, with limited communication and computation needed to compile the results and perform the necessary parameter updates (including inversions). We have implemented MPI and OpenMP parallelization (both can be used at the same time). As each focus pedigree forms an independent instance of the forward-backward algorithm, our approach can easily scale to as many threads as there are individuals. While we have only looked at it in proof-of-concept form, other work also shows that HMM implementations generally fare well in GPU parallelization [18].

### Comparison against existing approaches

Among existing work, our overall goal and approach is quite similar to Merlin. Merlin performs the full haplotype resolution of the full pedigree in a single iteration, but at the expense of an exponential state count explosion, which is somewhat mitigated by the use of sparse gene flow trees. Furthermore, the more efficient approximation mode in Merlin assumes the absence of more than some constant *t* recombinations taking place between any two adjacent markers (generally in the range [1,3]), with increasingly deteriorating performance for higher values of *t* (as the number of occurring states increases accordingly).

The most significant difference to our method, however, lies in the specific application of the model in order to determine haplotypes. Merlin uses a Viterbi decoding [7] for this purpose. This use might seem appropriate. The Viterbi algorithm gives the single most likely sequence of states matching the observations. The forward-backward algorithm we are using presents the probabilities for each state in each marker separately, conditioning on all observations, but not on the occupied states in other markers. If Viterbi training would be used in our approach, rather than Baum-Welch, the problem with local inversions could possibly be reduced, but at the cost of heavier bias towards initial interpretations. We are instead resolving that problem by the explicit inversion testing already discussed.

In the case of Merlin, however, the disadvantage in using the Viterbi algorithm is of a different nature, and lies in the construction of the state space, when used for genotype and haplotype inference. The state space distinguishes the phase in all individuals, even in homozygotic markers. The most likely *sequence of ordered allele pairs* for all individuals is thus different from the most likely *sequence of states*, as a single pair in the first can be split across multiple states (and individually thus be less likely) in the latter. When we reconstruct haplotypes, we want the former, but Viterbi will present a realization of the latter.

The sureness and skewness updates in our Baum-Welch-based approach are computing the posterior probabilities for precisely those variables. Hence, they do not suffer from this issue, although the underlying state space structure is similar.

Compared to our own earlier work, the main difference is that we have added the treatment of sureness, while previously our implementations only supported genotypes either being known (with a small constant allowing for errors), or completely unknown. As our method relies on information being transmitted between focus pedigrees through the iteratively updated parameters, the lack of parametrization of allele identity not only meant that parental genotypes could not be inferred, but also reduced the quality of haplotype inference in offspring, as linkage patterns between siblings could not be used directly.

### Materials

The first chromosome of the common dataset prepared for the 15th QTL-MAS workshop was used [19]. This dataset contains 3,220 individuals, containing 220 founders, 20 sires and 200 dams, with a uniform structure of 10 dams per sire, 15 offspring in each mating. 1,998 SNP markers were uniformly spaced on the chromosome with an intra-marker distance of 0.05 cM. The dataset for the QTL-MAS workshop is also suitable as it is supposed to reflect a situation of interest to animal and plant breeders alike. By using an externally simulated dataset, we avoid inadvertently tailoring our simulation approach to our method. By choosing smaller subsets from this dataset of relevant size, the data can approximate what would be expected from a set of multiple closely related case families for human disease association.

Comparisons were made against the Merlin package [4], which has performed favorably in other method comparisons [20]. When Merlin made ambiguous genotype calls, the first option was always chosen. As Merlin also tends to give uncertain calls for one of the two alleles at very high rates when the true genotype is homozygous, unknown alleles were replaced with the single identified allele in such loci. Preliminary studies showed that the added genotype errors introduced by this assumption were lower than the overall error rate. The version used was a pre-compiled binary of Merlin 1.1.2 with flags --best --horizontal --infer --bits 32.

Furthermore, to test parent genotype inference when genotyping calls in offspring were tested by artificially introducing an allele call error rate of 1.0*%* in offspring (approximately 2.0*%* genotype errors).

## Results

In all, we show that we can almost perfectly reconstruct parental genotypes, even when only fuzzy offspring genotype data is provided, given enough offspring individuals. General information on the experimental setting and our codebase is found in [6]. The computational resources used consisted of 8-core cluster nodes with Core i7 CPUs and 24 GiB of RAM, one node per job.

Final convergence of the model was studied by completing 150 iterations. The method was specified with 2 generations (4 states), as the dataset was limited to two generations.

### Subsets of full-sib families

**Figure 3:**
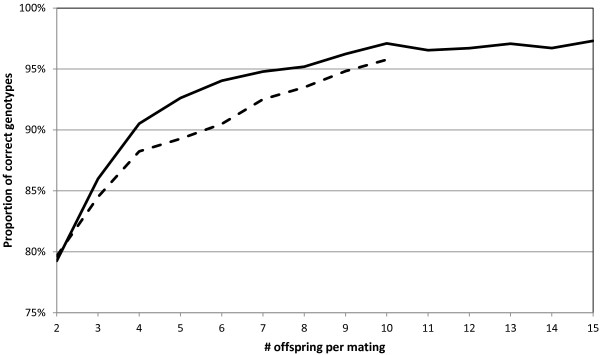
**Full-sib analysis.** Proportion of correctly reconstructed genotypes out of all markers, comparing cnF2freq (solid) with Merlin (dashed) for varying number of offspring per mating.

The case of reconstructing parental genotypes in full-sib families was analyzed by pruning the pedigree to only the first mating of each sire, with the first 2−15 offspring available. For up to 10 offspring, identical runs were executed using Merlin, after which we ran out of RAM for that tool due to the exponential increase in size of the state space, precluding analysis with larger subsets. The proportion of correctly identified genotypes out of all markers for the two tools is provided in Figure 3. Due to the inherent symmetry in full-sib structures, flips were done if necessary in order to align produced genotypes to original data for Merlin as well as for our code. Runs consumed between 60 and 110 minutes.

When both parents are homozygous with different alleles, phasing gives no additional information to aid genotype reconstruction (all offspring will be heterozygote). Our code can detect this and refuse to assign any genotype for those positions. Performance for correct genotype identification of those markers assigned, and the proportion of unidentified markers, can be found in Table 1.

**Table 1:** Correctly reconstructed genotypes in full-sib phasing for varying family size

**# offspring**	**Correctly phased**	**Markers ignored**
2	87.58*%*	14.49*%*
3	91.31*%*	9.53*%*
4	94.15*%*	6.93*%*
5	95.92*%*	6.36*%*
6	97.05*%*	5.83*%*
7	97.68*%*	5.75*%*
8	97.80*%*	5.37*%*
9	98.75*%*	5.14*%*
10	99.65*%*	5.14*%*
11	99.06*%*	5.14*%*
12	99.26*%*	5.14*%*
13	99.63*%*	5.14*%*
14	99.25*%*	5.14*%*
15	99.88*%*	5.14*%*

Proportion of incorrectly reconstructed genotypes out of those assigned, proportion of markers left unassigned.

### Analysis of full dataset

A full analysis was also performed over the complete dataset, where each sire participates in multiple matings, improving the power of genotype inference. The precision over all markers was 99.89*%*. For sires only, the precision was correct genotype for 99.98*%* of markers. The job consumed less than 450 minutes. Such an analysis over all families would be far beyond what is possible with models similar to the one used in Merlin, even if RAM would be expanded by several orders of magnitude. Therefore, we cannot compare this result to a suitable reference.

### Analysis with artificial errors in input data

Another full analysis was performed over the complete dataset, but with artificial genotyping errors at a rate of approximately 2.0*%* in offspring, as described in the Materials section. The current implementation does not update sureness values for the offspring, so the intent of the experiment was to verify to what extent the inferred parental genotypes were affected by the errors. In total, for all 220 founders, 99.51*%* of markers were inferred correctly. For the sires, the rate of correct genotypes was 99.97*%*. Runtimes were similar to the case with no introduced error.

## Discussion

The clear advantage of our approach is that much larger and more complex structures of closely related individuals can be analyzed simultaneously, allowing significant improvements in the precision of individual results. In one sense, our method is a decomposition of the full gene flow analysis performed in Merlin and similar tools, replacing the global analysis of all recombination events with parameters treating phase and genotype as probabilities. Despite this superficial limitation compared to Merlin, our methods produce superior results. A potential reason for this is that Merlin is using the Viterbi algorithm on the state space. The Viterbi algorithm selects the most probable state sequence, but as multiple states can map to identical haplotypes and genotypes, that is not necessarily the most likely genotype sequence. There are also numerical concerns when considering the very high number of states handled by Merlin, while our code has been explicitly designed to avoid or limit numerical inaccuracy. The use of skewness rather than explicit modelling of the full state hierarchy is one critical point in this regard.

We have also demonstrated that our approach to infer parental genotypes is resilient to genotyping errors, unlike trivial heuristics-based approaches as well as statistical approaches not modelling genotyping errors at all. For sires with a high number of offspring, the results are practically indistinguishable. Even when considering the parental population as a whole, the error rate in statistically inferred parental genotypes is markedly lower than the introduced error rate in offspring genotypes.

Our eventual goal is handling missing genotype data in complex multi-generational dense pedigrees in a general manner. For now, the codebase only updates sureness values in ancestors. Updates of sureness values to represent imputed genotypes for missing or unreliable data in offspring is a logical continuation of this work. Possibly, the initial data could be treated as a prior distribution for genotyping calls, rather than just initialization values. Otherwise, an optimization algorithm would prefer changing all genotypes into non-recombining representations of the parental haplotypes. Another natural extension would be to handle multiple generations. For the case of inferring haplotypes while treating genotypes as fixed, we have in earlier work demonstrated excellent results in complex pedigrees of 5 or more generations, with thousands of individuals. Due to the similarities in modelling, it is also reasonable to consider integration of our approach for pedigree data with Markov-model based phasing and imputation approaches for non-pedigreed individuals, such as MaCH [21].

Such a hybrid approach including Markov modelling in terms of non-related individuals would allow the model to gain information from haplotypes shared from historic ancestors beyond the known pedigree, thus handling population-level linkage disequilibrium (LD). In the current implementation, LD between unrelated individuals is not modelled explicitly, while a proper representation of recombination within families frequently makes this drawback one of limited consequence.

It would also be possible to first infer a local phasing for families with our method, followed by genotype imputation based on reference haplotypes from a denser map. Tools such as Minimac and some operation modes of other tools [22,23] expect the population where imputation is to be performed to already be pre-phased.

## Conclusion

We have presented an extension of our haplotyping approach for datasets with pedigrees and genotype data, to handle partially or completely missing ancestral genotype data efficiently. Haplotypes as well as genotypes can be inferred with high performance by using an efficient local Markov model, decomposing the pedigree into a set of focus pedigrees considering the direct ancestors to each individual. The decomposition is aided by iterative optimization of parameters that we call skewness and sureness, representing the unified information on phase and allele content from all focus pedigrees. We can analyze 150 closely related individuals with ease, providing near-perfect genotype and haplotype reconstruction, even for individuals where genotype was originally fully missing. In this setting, the otherwise comparable Merlin package tops out at 10 individuals.

Beyond the specific problem setting we have tested and our specific current implementation code, our results show that for phasing and genotype inference with Hidden Markov Models, it can be efficient to parametrize phase as well as allele content as scalars, allowing deterministic optimization schemes. Most existing work implementing some parametrization does this using simple binary variables accompanied by Markov-Chain Monte Carlo approaches (such as IMPUTE[13] and MaCH[14]). We are not aware of other work consistently implementing such a scalar parametrization for these types of problems.

## Availability and requirements

Our methodology has been included in a prototype implementation in the cnF2freq codebase, which has been extended to include the sureness parameter. The code is written in platform-independent C++. It is available from the project website, http://www.it.uu.se/research/project/ctrait/cnF2freq. The code is shared under a BSD-style license, meaning no restrictions regarding commercial use. It requires recent versions of the boost library [24], and has been tested with the Intel Compiler Collection version 11 and later, as well as the Gnu Compiler Collection version 4.2 and later.

Assistance in building the code, as well as necessary adaptations to handle specific requirements regarding input or output file formats, is available on request. A previous version of the base code is available with a standardized input format integrating with R [25] from https://r-forge.r-project.org/projects/cnf2freq/. The current additions can be added to this R package, on request.

## Competing interests

The author declares that he has no competing interests.

## References

[B1] Marchini J Howie B Genotype imputation for genome-wide association studies Nat Rev Genet 2010 11 7 499 511 10.1038/nrg2796 20517342

[B2] Lin S Chakravarti A Cutler D Haplotype and missing data inference in nuclear families Genome Res 2004 14 8 1624 10.1101/gr.2204604 15256514 PMC509272

[B3] Ding X Zhang Q Simianer H Haplotype Reconstruction and Estimation of Haplotype Frequencies from Nuclear Families with One Parent Available and Varying Numbers of Children Using the Exact Likelihood Human Heredity 2009 67 174 175 10.1159/000181155 19077435

[B4] Abecasis G Cherny S Cookson W Cardon L Merlin - rapid analysis of dense genetic maps using sparse gene flow trees Nat genet 2001 30 97 101 11731797 10.1038/ng786

[B5] Nettelblad C Holmgren S Crooks L Carlborg O cnF2freq: Efficient Determination of Genotype and Haplotype Probabilities in Outbred Populations Using Markov Models BICoB ’09: Proceedings of the 1st International Conference on Bioinformatics and Computational Biology 2009 Springer-Verlag, Berlin, Heidelberg 307 319

[B6] Nettelblad C Haplotype inference based on Hidden Markov Models in the QTL-MAS 2010 multi-generational dataset BMC Proceedings Volume 5 2010(Suppl 3) BioMed Central Ltd S10 S10 10.1186/1753-6561-5-S3-S10 PMC3103195 21624166

[B7] Rabiner LR A tutorial on hidden Markov models and selected applications in speech recognition Proceedings of the IEEE 1989 77 2 257 286 http://dx.doi.org/10.1109/5.18626 10.1109/5.18626

[B8] Lander E Green P Abrahamson J Barlow A Daly M Lincoln S Newberg L Newburg L MAPMAKER: an interactive computer package for constructing primary genetic linkage maps of experimental and natural populations Genomics 1987 1 2 174 10.1016/0888-7543(87)90010-3 3692487

[B9] Kruglyak L Daly MJ Reeve-Daly MP Lander ES Parametric and nonparametric linkage analysis: a unified multipoint approach Am j human genet 1996 58 6 1347 1363 http://view.ncbi.nlm.nih.gov/pubmed/8651312 8651312 PMC1915045

[B10] Therneau T Atkinson E Sinnwell J Schaid D McDonnell S kinship2: Pedigree functions. R package version 1.3.3 [http://CRAN.R-project.org/package=kinship2], 2011

[B11] Haldane JBS The combination of linkage values, and the calculation of distance between the loci of linked factors J Genet 1919 8 299 309

[B12] Broman KW Wu H Sen S Churchill GA R/qtl: QTL mapping in experimental crosses Bioinformatics 2003 19 7 889 890 http://bioinformatics.oxfordjournals.org/cgi/content/abstract/19/7/889 10.1093/bioinformatics/btg112 12724300

[B13] Howie BN Donnelly P Marchini J A Flexible and Accurate Genotype Imputation Method for the Next Generation of Genome-Wide Association Studies PLoS Genet 2009 5 6 e1000529 http://dx.doi.org/10.1371 10.1371/journal.pgen.1000529 19543373 PMC2689936

[B14] Li Y Willer CJ Ding J Scheet P Abecasis GR MaCH: using sequence and genotype data to estimate haplotypes and unobserved genotypes Genet Epidemiol 2010 34 8 816 834 http://dx.doi.org/10.1002/gepi.20533 10.1002/gepi.20533 21058334 PMC3175618

[B15] Baum LE Petrie T Soules G Weiss N A Maximization Technique Occurring in the Statistical Analysis of Probabilistic Functions of Markov Chains Ann Math Stat 1970 41 164 171 http://dx.doi.org/10.2307/2239727 10.1214/aoms/1177697196

[B16] Dempster A Laird N Rubin D Maximum likelihood from incomplete data via EM algorithm (with discussion) J R Statis Soc 1977 39 1 38

[B17] Seaton G Hernandez J Grunchec J White I Allen J De Koning D Wei W Berry D Haley C Knott S GridQTL: a grid portal for QTL mapping of compute intensive datasets Proceedings of the 8th World Congress on Genetics Applied to Livestock Production, Belo Horizonte 2006 MG, Brasil 13 18

[B18] Walters J Balu V Kompalli S Chaudhary V Evaluating the use of GPUs in liver image segmentation and HMMER database searches Parallel & Distributed Processing, 2009. IPDPS 2009. IEEE International Symposium on IEEE 2009 Rome, Italy, 1 12

[B19] Elsen JM Tesseydre S Filangi O Roy P Demeure O XVth QTLMAS: simulated dataset BMC Proc 2012 6 Suppl 2 S1 http://www.biomedcentral.com/1753-6561/6/S2/S1 10.1186/1753-6561-6-S2-S1 22640408 PMC3363151

[B20] Zhang K Zhao H A comparison of several methods for haplotype frequency estimation and haplotype reconstruction for tightly linked markers from general pedigrees Genet epidemiol 2006 30 5 423 437 10.1002/gepi.20154 16685719

[B21] Li Y Willer C Ding J Scheet P Abecasis G MaCH: using sequence and genotype data to estimate haplotypes and unobserved genotypes Genet epidemiol 2010 34 8 816 834 10.1002/gepi.20533 21058334 PMC3175618

[B22] Howie B Marchini J Stephens M Genotype Imputation with Thousands of Genomes G3: Genes, Genomes, Genet 2011 1 6 457 470 10.1534/g3.111.001198 PMC3276165 22384356

[B23] Huang J Ellinghaus D Franke A Howie B Li Y 1000 Genomes-based imputation identifies novel and refined associations for the Wellcome Trust Case Control Consortium phase 1 Data Eur J Human Genet 2012 20 7 801 805 http://dx.doi.org/10.1038/ejhg.2012.3 10.1038/ejhg.2012.3 22293688 PMC3376268

[B24] Boost C++ Libraries http://www.boost.org

[B25] The R Project for statistical computing http://www.r-project.org

